# Insights into the HyPer biosensor as molecular tool for monitoring cellular antioxidant capacity

**DOI:** 10.1016/j.redox.2018.02.023

**Published:** 2018-03-02

**Authors:** Helen Hernández, Alejandra Parra, Nicolas Tobar, Jessica Molina, Violeta Kallens, Miltha Hidalgo, Diego Varela, Jorge Martínez, Omar Porras

**Affiliations:** a Laboratorio de Biología Celular, Instituto de Nutrición y Tecnología de los Alimentos, Universidad de Chile, Chile; b Programa de Fisiología y Biofísica, Instituto de Ciencias Biomédicas, Facultad de Medicina, Universidad de Chile, Chile; c Millennium Nucleus of Ion Channels-Associated Diseases (MiNICAD), Universidad de Chile, Chile; d Centro de Investigación en Alimentos para el Bienestar en el Ciclo Vital (ABCvital), Universidad de Chile, Chile

**Keywords:** Antioxidant capacity, Biosensor, Fluorescence, Living cells, HyPer

## Abstract

Aerobic metabolism brings inexorably the production of reactive oxygen species (ROS), which are counterbalanced by intrinsic antioxidant defenses avoiding deleterious intracellular effects. Redox balance is the resultant of metabolic functioning under environmental inputs (i.e. diet, pollution) and the activity of intrinsic antioxidant machinery. Monitoring of intracellular hydrogen peroxide has been successfully achieved by redox biosensor advent; however, to track the intrinsic disulfide bond reduction capacity represents a fundamental piece to understand better how redox homeostasis is maintained in living cells.

In the present work, we compared the informative value of steady-state measurements and the kinetics of HyPer, a H_2_O_2_-sensitive fluorescent biosensor, targeted at the cytosol, mitochondrion and endoplasmic reticulum. From this set of data, biosensor signal recovery from an oxidized state raised as a suitable parameter to discriminate reducing capacity of a close environment. Biosensor recovery was pH-independent, condition demonstrated by experiments on pH-clamped cells, and sensitive to pharmacological perturbations of enzymatic disulfide reduction. Also, ten human cell lines were characterized according their H_2_O_2_-pulse responses, including their capacity to reduce disulfide bonds evaluated in terms of their migratory capacity.

Finally, cellular migration experiments were conducted to study whether migratory efficiency was associated with the disulfide reduction activity. The migration efficiency of each cell type correlates with the rate of signal recovery measured from the oxidized biosensor. In addition, HyPer-expressing cells treated with N-acetyl-cysteine had accelerated recovery rates and major migratory capacities, both reversible effects upon treatment removal. Our data demonstrate that the HyPer signal recovery offers a novel methodological tool to track the cellular impact of redox active biomolecules.

## 1 Introduction

Antioxidant consumption is a widely acquired nutritional behavior. Underlying the justification of this conduct is the promise that high antioxidant intake prevents oxidative damage, thus avoiding a variety of diseases associated with an intracellular redox imbalance [1], [2].

The antioxidant property of some molecules is currently assessed by evaluating their capacity to protect a target molecule from oxidation in an abiotic environment [3]. Several constraints arise to the use of this widespread analytical process. First, the antioxidant property is evaluated without considering cells (i.e., far from interactions with cellular components that affect the reactivity of the tested molecule). Second, the chemical-based procedure to analyze antioxidant properties consists of merely exposing the target molecule to a unique interaction with the antioxidant; a nonexistent situation in the intracellular environment, where antioxidants must deal with a variety of cellular components that can exert a collaborative effect with the tested molecule. And third, by their nature, antioxidant chemical analyses are limited to a reduced time of action, even though the antioxidant inside the cellular environment is exposed to many sequential reactions over a longer time, and with the chance of affecting long-term cellular defense mechanisms.

The combination of molecular biology and imaging techniques has created many genetically-encoded fluorescent biosensors to follow intracellular parameters in living cells, overcoming the majority of the limitations related with redox chemical analyses depicted above [4]. Typically, a biosensor is a fusion protein composed by a motif that recognizes a specific molecule, usually taken from bacteria, and assembled with one or two fluorescent proteins. Upon binding of the target molecule, a conformational change is induced that modifies the spectral properties of the fluorescent domains. Some sensors have two fluorescent domains with overlapping emission and excitation spectra, capable of undergoing Förster resonance energy transfer (FRET) and responding to the molecule with a change in FRET efficiency [5].

A biosensor for tracking intracellular hydrogen peroxide in living cells was developed in 2006 by Lukianov et al. [6]. The probe, HyPer, consists of a circularly permuted yellow fluorescent protein (cpYFP) inserted into the regulatory domain of the *Escherichia coli* H_2_O_2_-sensing protein OxyR. The properties of wild type OxyR, such as specificity and sensitivity to H_2_O_2_, are conserved in this tool, which has been useful to detect H_2_O_2_ variations under growth factor stimulation in mammalian cells [7] and to resolve spatial and temporal oxidation events in Tau-HyPer HeLa cells [8]. As bacterial OxyR, HyPer biosensor does not respond to oxidized molecules derived from nitrosative stress. Although S-Nitrosothiols are able to react with Cys-199 at OxyR [9], the reaction does not proceed to form a disulfide bond [10], a requirement to achieve conformational change in the biosensor, which limits the capacity of HyPer to monitor nitrosative stress events. Despite of this limitation, this molecular tool allows overcoming one of the main disadvantages of dichlorofluorescein (DCF) based methods to measure cellular reactive oxygen species (ROS), since DCF produces ROS under light exposure [11].

The use of HyPer has been concentrated in measuring levels of H_2_O_2_ in living cells under receptor activation and fisiopathological conditions both in mammalian [12], [13], [14] and plant cells [15], [16], [17]. Intracellular H_2_O_2_ levels are driven primarily from enzymatic reductions of superoxide anions, carried out by superoxide dismutases (SODs). Mitochondrial metabolism is one of the main contributors of superoxide anion production, specifically as a marginal electronic leakage through the electron chain transport [18]. Less contributing processes, which deal with electron transference, are xanthine oxidases and NADPH oxidase, among others. All these observations have helped to conceive a heterogeneous spatio-temporal distribution of intracellular H_2_O_2_ according to the subcellular site of generation, the nature of triggering stimulus and the abundance of antioxidant systems dedicated to neutralize peroxide excess [18], [19], [20], [21].

On the other hand, much less attention has been put in the cellular processes involved in the recovery of oxidized HyPer. Disulfide bond reduction formed on proteins is probably carried out by Thioredoxin Reductase/thioredoxin or Glutathione Reductase systems. In this line, it has been proposed that Hyper reduction could be a useful method to assess the antioxidant activity in living cells [19], [20]. In this study, we have analyzed HyPer signal during and after the application of pulses of hydrogen peroxide in order to dissect biosensor responses. Recovery signal rates from an oxidized state discriminated better the reducing properties of three subcellular compartments (cytosol, mitochondrion and endoplasmic reticulum) than steady-state measurements. Our study includes pH dependency of HyPer parameters as a baseline, the response to H_2_O_2_ and the recovery from an oxidized state on pH-clamped cells, a technique that conserves cellular integrity and ensures intracellular pH control.

Our results allowed us to gain information about the antioxidant capacity of the cytoplasmic environment in terms of the efficiency of counterattacking an incoming oxidant bolus and the velocity to restore disulfide bonds formed on the biosensor. We also connected the biosensor signal recovery rate with the migration potential by determining that cell lines that presented the highest recovery HyPer rates also had the highest migratory capacity. Moreover, we observed that by increasing the reductive tone of cells by adding an exogenous cysteine donor, like N-acetyl-cysteine, also resulted in a major migratory capacity. Our results indicate that HyPer recording can become a useful tool for reporting changes in antioxidant capacity in the environment and is suitable for evaluating the cellular impact of compounds with antioxidant potential.

## 2 Materials and methods

Reagents and salts for buffer preparation were purchased from Sigma-Aldrich (MO, USA). Lipofectamine® 2000, media culture and supplements were acquired from Life Technologies (NY, USA). EUK-134 was purchased from Cayman Chemical Company (MI, USA). PX-12, nigericin, and valinomycin were acquired from Tocris (MO, USA). BCECF-AM was acquired from Thermo Fisher Scientific (MA, USA). The 30% hydrogen peroxide solution used in this work was acquired from Merck (Darmstadt, Germany).

### 2.1 Cell culture

All cell lines were purchased from the American Type Culture Collection (ATCC): HTB-45™, epithelial cells from human kidney adenocarcinoma (A704); HB-8065™, epithelial cells from human hepatocellular carcinoma (HepG2); CRL-8621™, astroglia derived from human fetus brain corresponds to SV40 transformed cells (SVGp12); HTB-81™, brain metastatic epithelial cells derived from human prostate carcinoma (DU145); CRL-2086™, human fibroblasts derived from breast carcinoma (CCD-1068SK) and HTB-37™, epithelial cells from human colorectal adenocarcinoma (Caco-2) were cultured in MEM-supplemented 10% fetal bovine serum. CCL-18™, epithelial cells from lung carcinoma (A549) required F-12K Medium, supplemented with fetal bovine serum to a final concentration of 10%. HTB-22™, epithelial cells from pleural effusion derived from human mammary gland adenocarcinoma (MCF-7) were cultured in DMEM/F12 supplemented with 10% fetal bovine serum. Ad-293 cells, derived from HEK-293 cells with improved adherence properties, were kindly donated by Dr. Diego Varela (Universidad de Chile) and were cultured in Eagle's Minimum Essential Medium, supplemented with 0.01 mg/ml human recombinant insulin and 10% fetal bovine serum. CRL-2310™, keratinocytes from human fetus correspond to papillomavirus transformed cells (CCD-1102 KERTr) were grown in Keratinocyte-Serum-Free Medium, with added keratinocyte supplements, including bovine pituitary extract (Gibco, TermoFisher Scientific, USA) and human recombinant epidermal growth factor (Gibco, TermoFisher Scientific, USA), further supplemented with an additional 30 ng/ml human recombinant epidermal growth factor (BD Biosciences, USA). CRL-4025™, endothelial cells from neonatal dermic microvasculature (TIME) were cultured in Vascular Cell Basal Medium, supplemented with the Microvascular Endothelial Cell Growth Kit-VEGF and 12.5 μg/ml blasticidine. In general terms, cells were maintained under a humidified atmosphere with 5% CO_2_/air, and culture media was renewed every 2 or 3 days. When 70 – 80% of confluence was reached, cultures were expanded to other plaques or seeded on glass coverslips to be further imaged. At this point, it is necessary to state that all cell types were used within passage numbers 3–15 where no significant changes in baseline values were found (data not shown).

### 2.2 Adenoviral particles production and infection

Adenoviral vectors were generated using the AdEasy system [21]. Briefly, cyto-HyPer cDNA (Evrogen, Moscow, Russia) was sub-cloned into the commercial adenoviral vector pAdEasy-RFP using conventional molecular biology techniques, while homologous recombination was performed using BJ5183 cell transformation. Recombinant adenoviral plasmids were transfected in Ad-293 cells with lipofectamine, following manufacturer guidelines. Following observation of cytopathic effects (CPE), usually after 14–21 days, cells were harvested and subjected to three freeze-thaw cycles, followed by centrifugation to remove cellular debris. The resulting supernatant (2 ml) was used to infect a 10 cm dish of 90% confluent Ad-293 cells. Following CPE observations after 2–3 days, viral particles were purified and expanded by infecting 10 plates of AdHek cells. Finally, target cells were grown on 25 mm coverslips to be infected by adding a 1:500 virus dilution. Two days later, cells were ready for HyPer imaging.

### 2.3 HyPer imaging

On the experimental day, the coverslips were mounted in an open recording chamber and media were replaced with KRH buffer (in mM: 140 NaCl, 4.7 KCl, 20 Hepes, 1.25 MgSO_4_, 1.25 CaCl_2_, pH 7.4), supplemented with glucose 5 mM. Imaging took place in an inverted Nikon Ti Eclipse microscope equipped with 40X oil objectives [numerical aperture, N.A. 1.3]. A Xenon lamp was coupled to the monochromator device (Cairn Research Ltd, Faversham, UK). Digital images were acquired by means of a cooled CCD camera (Hamamatsu ORCA 03, Japan). Devices synchronization and technical adjustments were under analogue-digital interfase provided by micromanager software [22].

Before time-lapse started, exposure time to each channel (420 and 490 nm) was adjusted to obtain a satisfactory signal-to-noise ratio, usually 50–200 ms. Emitted fluorescence for each excitation channel was achieved by a long-pass filter over 520 nm. In order to obtain a ratiometric measurement, exposure time was the same for each excitation channel and constant during the whole experiment. Fluorescence from any single cell was quantified by drawing the same ROI (region of interest) in images derived from both channels.

Upon hydrogen peroxide exposure, biosensor signals responded reciprocally as expected, showing a reduction in the signal, corresponding to excitation at 420 nm, along with a simultaneous increment in the signal obtained at 490 nm (Supplementary Fig. 1A). Both excitation wavelengths were acquired each 20 s and expressed as a ratio (490 nm/420 nm), making H_2_O_2_ recordings reliable and independent of the biosensor expression. For every record, an initial lapse of 20 min was done to ensure a reliable and stable baseline, denominated basal value. Then, two consecutive pulses of hydrogen peroxide were applied. The first was of 50 μM; enough to evoked a moderate and transient increase in HyPer ratio. The second was a saturating pulse of 500 μM of H_2_O_2_; useful to determine the maximal response of HyPer (Supplementary Fig. 1B and C). Using this strategy, we could record the kinetics of the rising HyPer signal at the first pulse and signal recovery following the saturating pulse (Supplementary Fig. 1B). In order to compare rising and recovery rates from the recordings obtained from different cell types and experimental conditions, we normalized the change in the biosensor ratio using the maximal change recorded between the basal and saturating pulse to assign the response range of the biosensor. The plasmid used to target HyPer to the mitochondria was pHyPer-dmito (Evrogen, Moscow, Russia). The plasmid carrying the KDEL signal that sends HyPer to the endoplasmic reticulum (ER) was donated by Dr. Claudio Hetz from the Universidad de Chile. The targeting of the indicated subcellular compartment by the biosensor was consistent with the pattern of fluorescent signal obtained from living cells (Supplementary Fig. 2).

### 2.4 Intracellular pH imaging

Cells grown on coverslips were mounted in an open recording chamber and media was replaced by KRH buffer supplemented with 5 mM glucose. Cells were loaded with 5 μM of the pH sensitive dye BCECF for 30 min. Then, cells were rinsed three times with KRH and left at room temperature for another 10 min to allow the remnant BCECF-AM molecules to be de-esterified. For pH imaging, excitation at 440 and 490 nm took place simultaneously and the emitted light was collected by a long-pass filter over 520 nm. This setting permits ratiometric imaging of intracellular pH. Intracellular pH was also monitored by transfecting plasmid SyPher (#48250 donated by Nicolas Demaurex, Addgene, USA) with lipofectaine 2000®, following the provider's instructions. This plasmid carries the HyPer sequence with a single mutation in C199S, resulting in a biosensor unable to sense H_2_O_2_ but responding to variations in pH [23]. In order to clamp intracellular pH, a mixture of valinomycin and nigericin was used together with a high K^+^ buffer, equivalent to the KRH buffer described above, in which Na^+^ was equimolarly replaced by K^+^. In both types of recordings (i.e., using the BCECF dye or the SyPher biosensor), data was acquired over 20 s and expressed as 490 nm/440 nm ratio for BCECF and 490 nm/420 nm ratio for SyPher.

### 2.5 Cell migration assay

Cell migration was studied using a 6.5 mm Transwell chamber with an 8 µm pore size (Corning Inc., MA, USA). Transwell membranes were seeded with 6×10^4^ cells in serum-free medium on the upper compartment of the chamber. Cells were allowed to migrate for 8 or 16 h against a gradient of 2% FBS. After this, membranes were fixed with a solution of 0.2% crystal violet in methanol. Cells on the upper membrane surface were then removed using a cotton swab and repeatedly washed with water to remove crystal violet excess. Migratory cells on the lower side of the membrane were counted by observing four fields per chamber at 20X objective [24].

### 2.6 Statistical analysis

Throughout the manuscript, data are expressed as mean±standard error. Paired Student's *t*-tests were executed only for comparisons of “before-after” experimental designs, whereas comparisons of multiple groups or repeated measurements were evaluated by ANOVA with Bonferroni post-hoc analysis for parametric data or Dunn's method for non-parametric data. A p-value less than 0.05 was considered statistically significant. It is important to mention that cellular imaging allowed us to execute single-cell recordings, meaning that several cells were monitored in each experiment. This means that data for each experimental condition was achieved by pooling data from at least three independent experiments.

## 3 Results

### 3.1 HyPer signal recording in living cells subjected to transient oxidative challenges

**Fig. 1:**
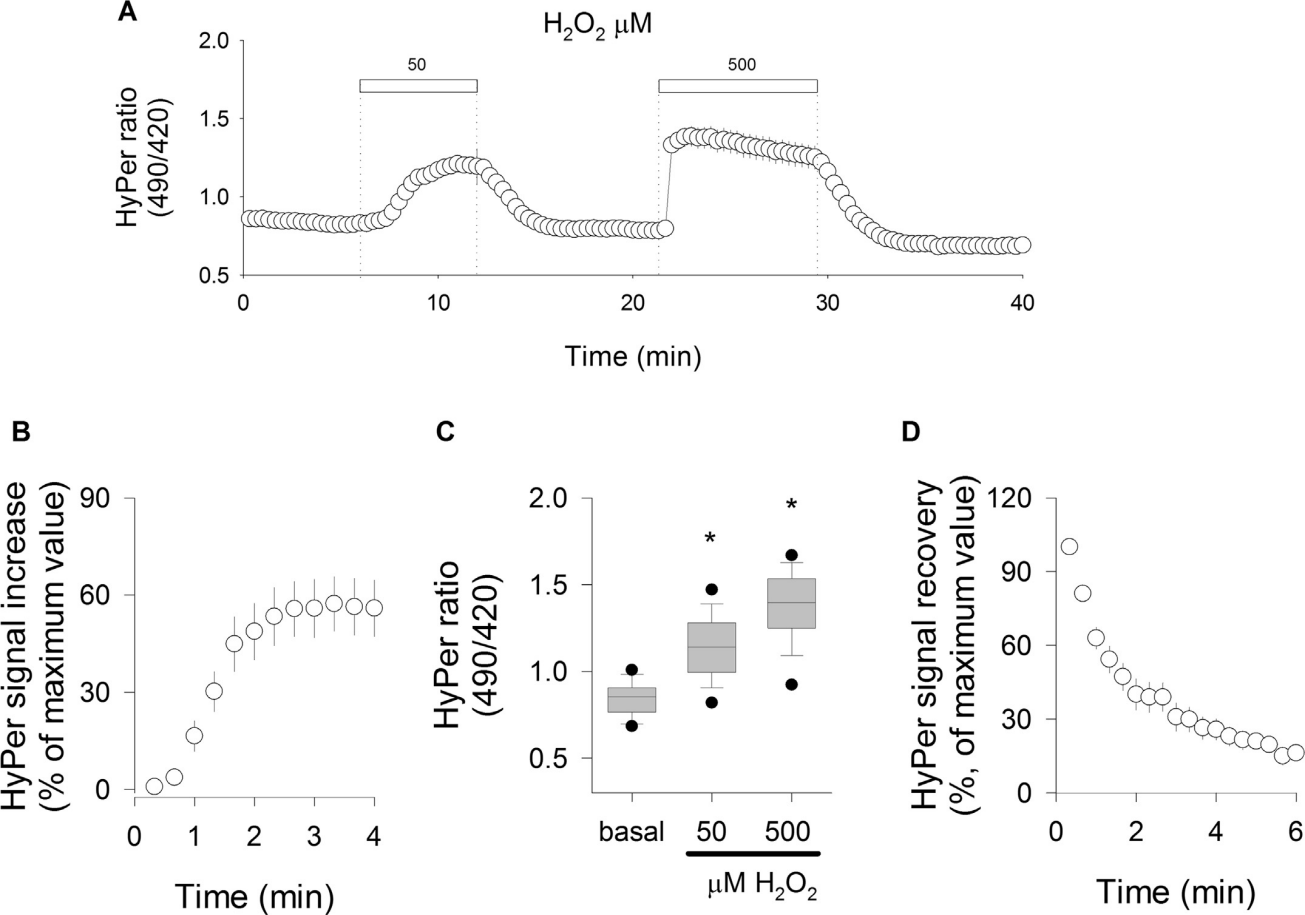
Recording and analysis of ratiometric HyPer signal in TIME cells. A. Typical recording of the biosensor response to two consecutives pulses of H_2_O_2_ in TIME cells. Pulses were applied at the time indicated by the white bars and dotted lines. Data correspond to averages±SE of 13 cells from one representative experiment. B. Time-course of HyPer signal increasing during the first 4 min of 50 µM H_2_O_2_ application. Data are shown as percentages with respect to the maximal value obtained with 500 µM H_2_O_2_. C. Comparison of the basal and maximal signal responses obtained at 50 µM and 500 µM H_2_O_2_ pulses; data are represented with boxes, where the middle lines indicate median values. Asterisks over the boxes indicate significant differences compared to basal values (Kruskal-Wallis ANOVA, Dunn's Method). D. Time-course of HyPer signal recovery immediately after wash out with the 500 µM H_2_O_2_ pulse; data are shown as percentages with respect to maximal values obtained with the 500 µM H_2_O_2_ pulse. In plots B, C, and D, data correspond to averages±SE of 43 cells from four independent experiments.

To test the efficacy of the molecular sensor in responding to changes in the intracellular oxidative tone, we transduced endothelial TIME cells with an aliquot of an adenovirus expressing the HyPer biosensor, as described in Materials and Methods. Forty eight hours after transduction, sensor-expressing cells excited at 420 and 490 nm rendered a stable baseline signal (Fig. 1**A)**. When HyPer-expressing cells were exposed to hydrogen peroxide in a modality of pulses (50 and 500 μM), transient HyPer signal responses were consistently observed. First, a gradual increase in the biosensor ratio was observed, which reached a plateau after 3 min of H_2_O_2_ addition (Fig. 1**B**). This kinetic behavior was useful to obtain reliable values for biosensor responses from several recordings for submaximal (50 μM) and maximal responses (500 μM), as depicted in Fig. 1**C**. After removing the oxidant pulses, a spontaneous recovery of HyPer signal was observed (Fig. 1**A and D**). Finally, the addition of the saturating pulse of 500 μM hydrogen peroxide induced a faster oxidation of the biosensor compared with previous H_2_O_2_ pulses. Note that the maximum signal increment occurred within 20 s, which corresponds to the sampling frequency of the images obtained. The removal of the 500 μM pulse led to a gradual decline in the signal, which corresponded to the spontaneous recovery of the biosensor (Fig. 1**D**).

### 3.2 Hyper biosensor reports differential redox properties at three different subcellular compartments

**Fig. 2:**
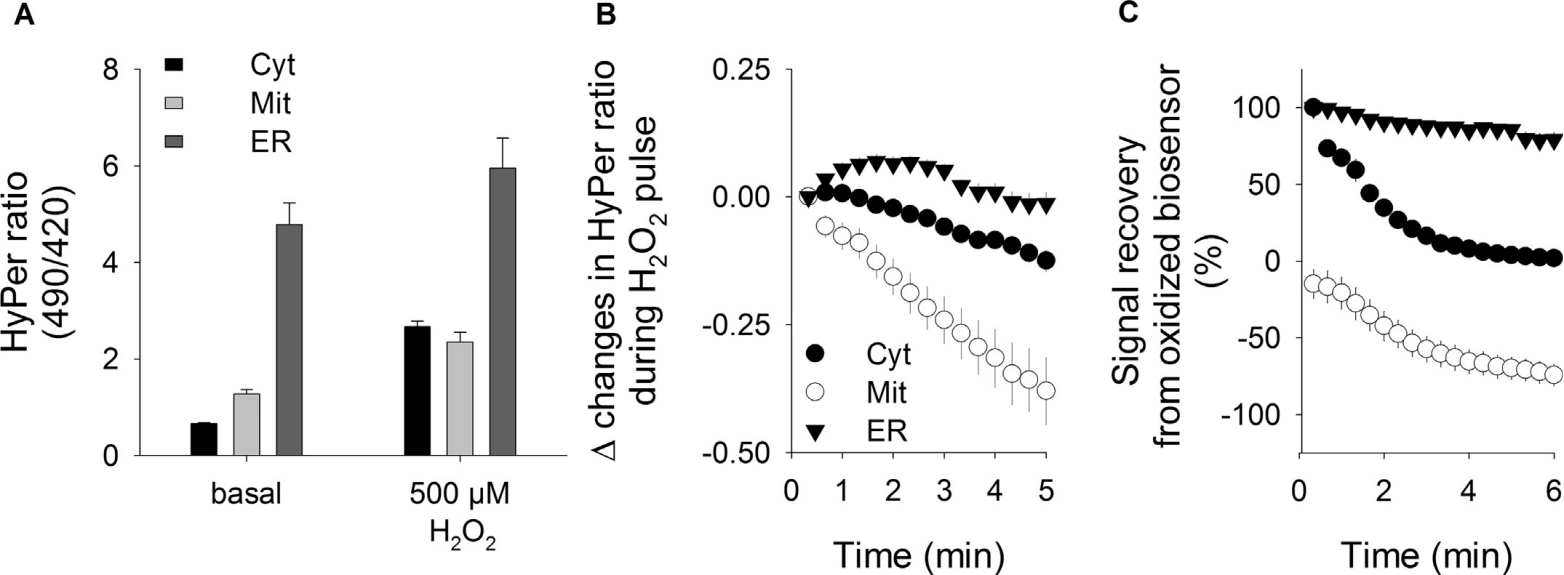
HyPer biosensor reports the local antioxidant capacity at three subcellular compartments. HyPer biosensor was targeted on the cytoplasm (Cyt), mitochondrion (Mit), and endoplasmic reticulum (ER) via the transfection of Ad-293 cells with the corresponding plasmids, as described in Materials and Methods. Two days after transfection, the cellular imaging experiment took place. A. HyPer signal before (basal) and after a single pulse of 500 μM H_2_O_2_ collected from Ad-293 cells expressing the biosensor in the cytoplasm (black bars), mitochondrion (light gray bars) and endoplasmic reticulum (dark gray bars). B. Time-course of HyPer signal in the presence of 500 μM H_2_O_2_ recorded in the cytoplasm (black circles), mitochondrion (open circles) and endoplasmic reticulum (black triangles). Data are presented as the change between the ratio to the point when the H_2_O_2_ pulse was applied and the subsequent points. **C**. Recoveries of the HyPer signal recorded at these three subcellular compartments obtained after wash out of the 500 μM peroxide pulse. Maximal signal reached by 500 μM was considered as full oxidized biosensor and data was expressed as percentage of this maximal value. Data correspond to averages±SE of 27–51 cells from at least four independent experiments.

To analyze the biosensor responses at different subcellular environments, we used Ad-293 cells to express HyPer onto three cellular compartments with well-defined redox properties: cytoplasm, mitochondrion and endoplasmic reticulum (ER). HyPer in ER showed the highest basal signal, which is consistent with the oxidative tone associated with proper protein folding function associated to this organelle. Upon the exogenous application of 500 μM hydrogen peroxide, the ER signal experienced an increase from basal value of roughly 25%, whereas the same oxidative pulse increased the HyPer signal by 300% and 85% in the cytoplasm and mitochondrion, respectively (Fig. 2A). In the presence of the hydrogen peroxide pulse, we observed that the HyPer signal from mitochondria showed a clear tendency to diminish, even in the presence of the oxidant agent in the extracellular space. The other two compartments showed small variations in HyPer signal (Fig. 2B). This compartment-dependent behavior of HyPer kinetics was also noticeable following hydrogen peroxide wash out, where HyPer signal recovery occurred by intrinsic machinery located at the compartments evaluated. For HyPer located at ER and cytoplasm, the biosensor signal recovery was possible to be recorded since the biosensor still remained in its oxidized form, whereas in mitochondrion the biosensor was almost completely reduced back to its basal level even in the presence of H_2_O_2_. Recovery signal kinetic from ER was gradual and slower compared to the signal recovery recorded in the cytoplasm (Fig. 2C). These observations cannot be explained only by differences in the dynamic range of the HyPer biosensor in the subcellular environments presented here, since the H_2_O_2_ responses between mito-HyPer and ER-HyPer are comparable (mito: 0.93 ± 0.07 and ER: 1.13 ± 0.15), while the kinetic behavior between these compartments are totally different.

### 3.3 Recovery phase of HyPer signal is a pH independent parameter

**Fig. 3:**
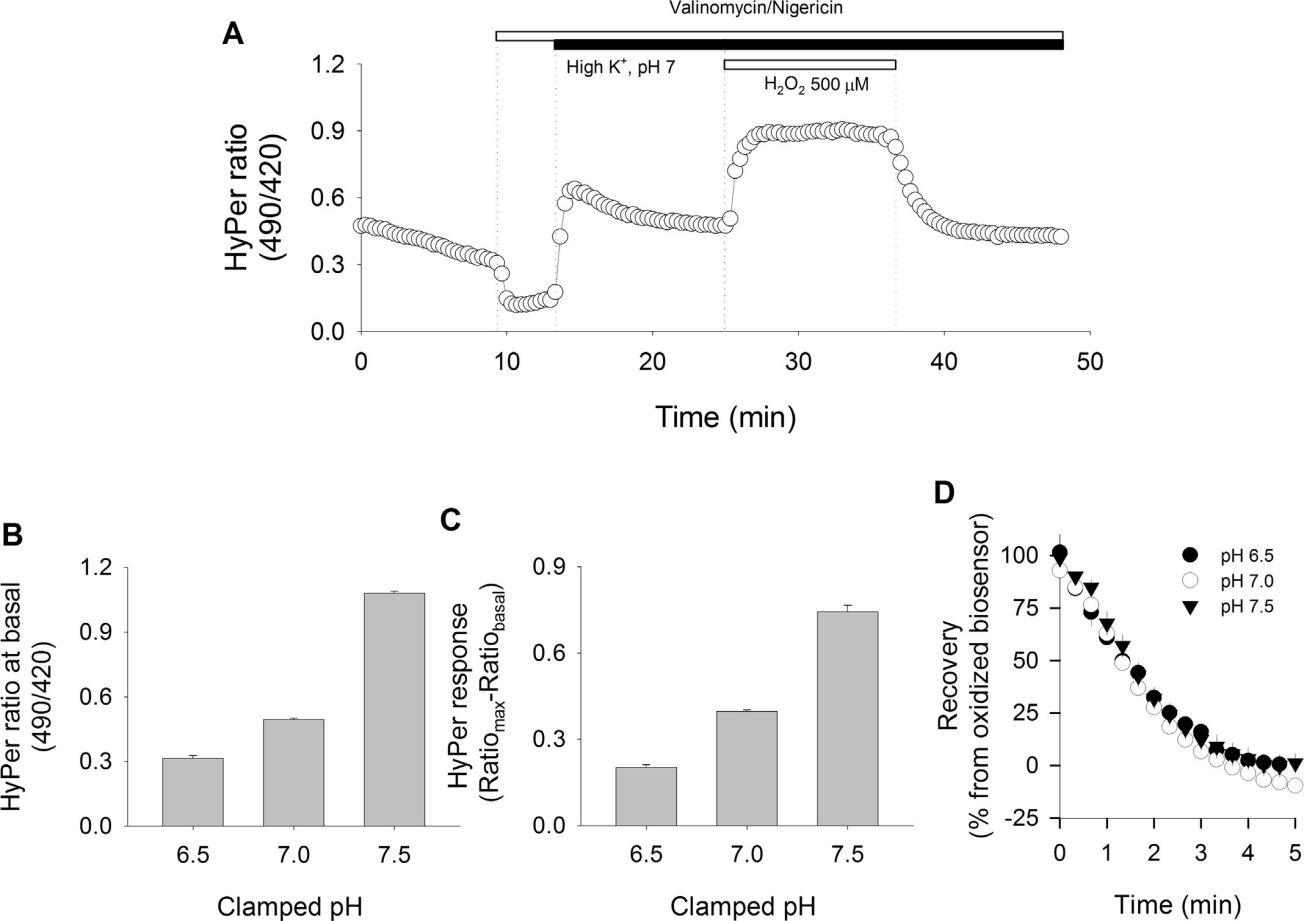
HyPer signal recovery is independent of pH. A. TIME cells expressing the HyPer biosensor were subjected to pH clamping by using a mixture of nigericin/valinomycin (100 μM/25 μM), as indicated by the horizontal white bar and the dotted line. After that, normal KRH buffer was replaced by a high K^+^ buffer at pH 7.0 (black bar). Once equilibrium was reached, cells were transiently exposed to 500 μM H_2_O_2_ (gray bar). Data correspond to averages±SE of 15 cells from a representative experiment. B. Comparison of basal values for HyPer at different pH values obtained by averaging ratio values 2 min before H_2_O_2_ pulse addition. C. Responses of HyPer biosensors are expressed as the difference between the maximal value obtained during the 500 μM H_2_O_2_ pulse and the basal value at the pH values indicated. **D.** Comparisons of HyPer signal recovery kinetics were possible by taking data immediately following H_2_O_2_ pulse wash out at pH 6.5 (black circles), pH 7.0 (open circles) and pH 7.5 (black triangles). For B, C and D, data correspond to 31, 32, and 23 cells, respectively, and each experiment was repeated at least three times.

HyPer biosensor responses are influenced by environmental pH [6]. Here, we analyzed three parameters of the biosensor behavior: at baseline, its response to 500 μM H_2_O_2_ and its recovery rate, all in cells subjected to intracellular pH clamp using the nigericin/valinomycin technique [25]. This technique consists in exposing living cells to a mixture of these two ionophores along with a physiological extracellular solution containing high K^+^ concentration with a defined pH. Increment of extracellular K^+^ concentration ensures rapid equilibrium through valinomycin and efficient H^+^/K^+^ interchange by nigericin pores in the plasma membrane, allowing the extracellular pH to quickly reach equilibrium with the intracellular compartment. The effectiveness of controlling intracellular pH was assessed by monitoring in real-time the intracellular pH changes with the BCECF dye and by using SyPher, a mutated version of HyPer that is sensitive to pH but insensitive to H_2_O_2_ (**see** Supplementary Fig. 3). Fig. 3A shows an example of HyPer recording in TIME cells which were subjected to pH clamp technique. Using this protocol, we observed the large variation in baseline values as well as in the responses to H_2_O_2_ that HyPer elicits at three physiological relevant pH values (Fig. 3B and C). However, analysis of the HyPer signal recovery showed similar rates at the evaluated pH values (50.7±3.1 min^−1^ for pH 6.5; 50.2±5.5 min^−1^ for pH 7.0 and 52.3±7.1 min^−1^ for pH 7.5). This finding places this kinetic parameter as a more reliable tool for quantifying the total antioxidant activity of the intracellular environment (Fig. 3D). As expected, addition of H_2_O_2_ to cells, either before or after application of the nigericin/valinomycin mixture, did not induce changes in the SyPher signal (data not shown).

### 3.4 Recovery HyPer signal is sensitive to pharmacological manipulation of the cellular antioxidant machinery

**Fig. 4:**
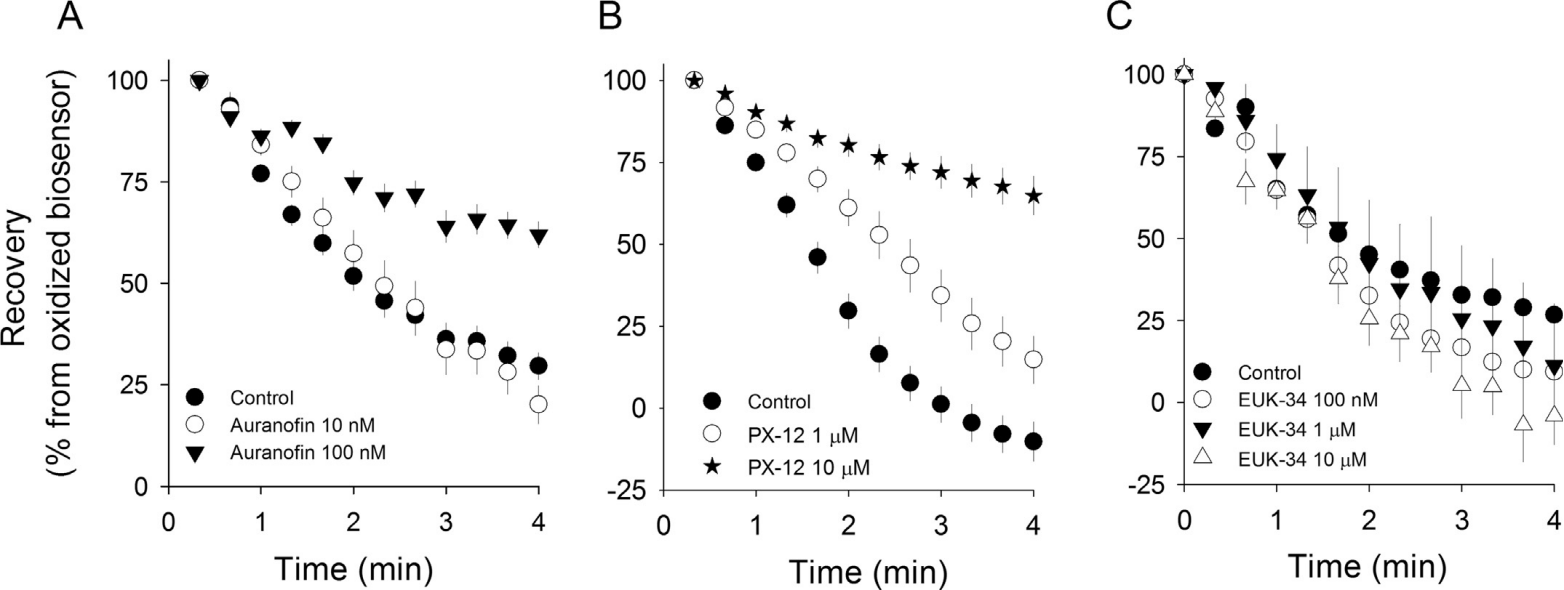
HyPer signal recovery was impaired by selenium chelation and pharmacological inhibition of thioredoxin-1 but was insensitive to SOD-catalase mimetic, EUK-134. Two days following infection with adenovirus, TIME cells expressing the biosensor were treated with (A) auranofin for 24 h, (B) PX-12 for 10 min and (C) EUK-134 for 24 h. Plotted traces correspond to the recovery phase of the HyPer signal after wash out with a pulse of 500 μM H_2_O_2_. A. TIME cells in control condition (Ctrl, black circles) or treated with 10 nM (Au 10 nM, open circles) and 100 nM (Au 100 nM, black triangles) of Auranofin. B. Comparison of the recovery HyPer signal after wash out with 500 µM H_2_O_2_ pulse from TIME cells at control conditions (black circles) or treated with 1 µm (black stars, 26 cells from three independent experiments) or 10 µM (open circles; 48 cells from five independent experiments) PX-12 for 24 h. C. Control (30 cells, from four independent experiments), 100 nM EUK-134 (27 cells from three independent experiments), 1 μM EUK-134 (37 cells, from four independent experiments), 10 μM EUK-134 (32 cells, from three independent experiments). In order to estimate decay rates from HyPer recovery curves, data were fitted to a single decay exponential function of three parameters.

The above results related to HyPer signal recovery suggested that it was feasible to track the activity of the intrinsic antioxidant machinery expressed in living cells. To test this concept, and to identify molecular candidates involved in this activity, we incubated TIME cells for 24 h with auranofin, a selenium quelator that disrupts the proper assembly of selenocysteine redox- enzymes (including glutathione peroxidase and thioredoxin reductases). With this treatment, cells maintained their morphology with no evident signs of cellular stress. Basal HyPer values were similar in the groups of treated cells (Supplementary Fig. 4), but the recovery rate decreased from 41.8 ± 1.1 min^−1^ in control cells to 13.7±1.0 min^−1^ in cells treated with 100 nM auranofin, while no effect was evident with 10 nM auranofin (Fig. 4A). Similarly, the rate of HyPer signal recovery measured in TIME cells exposed to PX-12, a pharmacological inhibitor of thioredoxin-1, diminished from 88.5 ± 10.7 min^−1^ in control cells to 38.8±6.2 and 25.8 ± 3.7 min^−1^ in cells treated with 1 μM and 10 μM PX-12, respectively (Fig. 4B). PX-12 was applied only when hydrogen peroxide was washed out, since incubation with this compound for prolonged times produces blebs in the plasma membrane and further cellular collapse. Additionally, cellular treatments with 1 μM and 10 μM EUK-134, a synthetic compound that combines superoxide dismutase and catalase activities, effectively rendered lower levels of basal H_2_O_2_ in treated versus untreated cells (Supplementary Fig. 4). We observed no effect on HyPer recovery curves compared to control cells (Fig. 4C). Thus, these results strongly suggest that the cellular disulfide-bond reducing activity is composed, at least in part, by selenoenzyme systems and cytoplasmic thioredoxin-1. In contrast, treated cells with a catalase/SOD mimetic had no effect in the cellular reducing capacity.

### 3.5 Spectrum of differences according to HyPer biosensor responses on different human cell lines

**Fig. 5:**
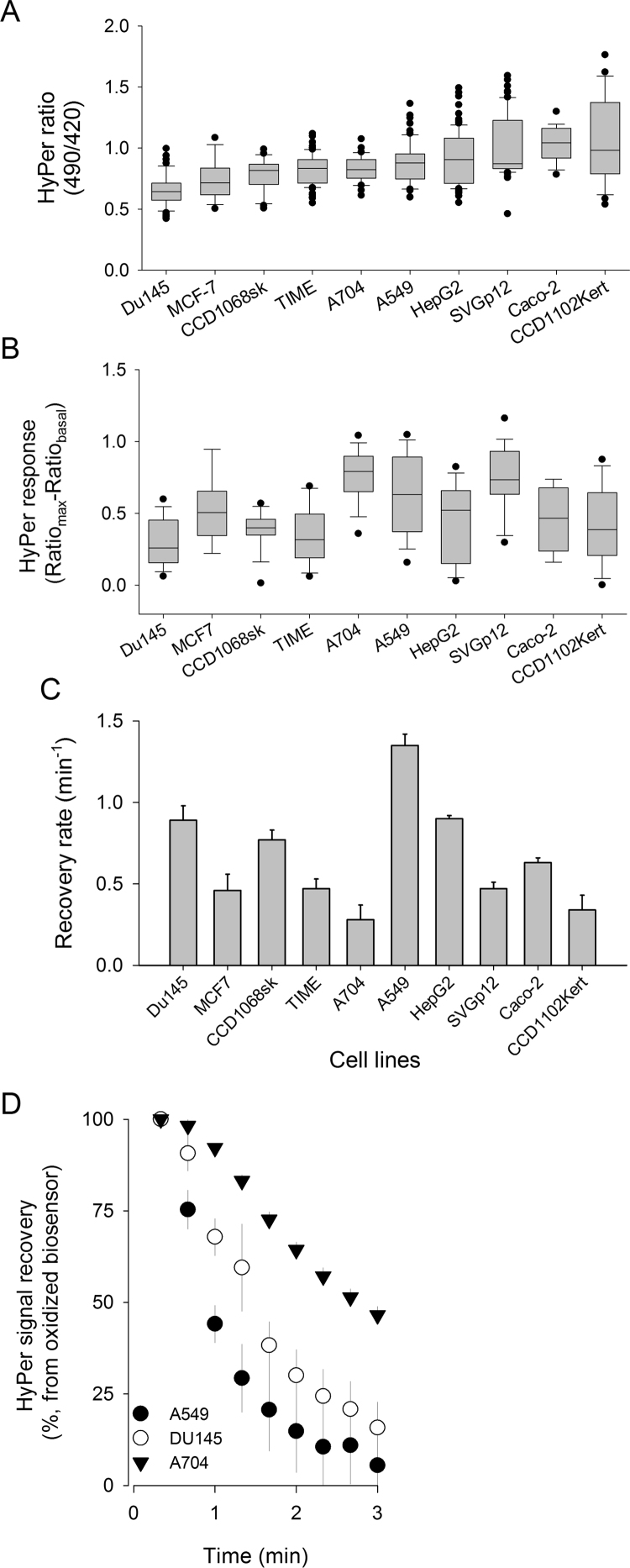
Analysis of HyPer signal on several human cell lines. A. Basal signal of HyPer obtained from different cell line cultures, as indicated in Materials and Methods, displayed from the lowest to the highest values. B. Response of HyPer after the exposure to 50 μM H_2_O_2_. Data were obtained measuring the maximum response upon stimulus for each individual cell, with respect to its corresponding basal value. C. Spontaneous recovery rates from an oxidized biosensor were estimated from experimental data recorded after removal of 500 μM H_2_O_2_ pulse. D. Time course of the recovery phase of oxidized biosensor in three cell types: A549 representing the fastest recovery, DU145 representing a mild recovery and A704 being slowest cell line to recover. Data correspond to averages±SE of more than 30 single cell recordings obtained from three or more independent experiments.

To test whether HyPer biosensor is capable to detect differences in the cytoplasmic environments from different cell types, we expressed HyPer in ten human cell lines originated from a variety of human tissues and performed the following analyses: the basal signal of HyPer as a steady state parameter defined by endogenous H_2_O_2_ production and the cellular capacity to remove it and establish its characteristic reducing tone (Fig. 5A); the response of the biosensor, expressed as the difference between the maximum ratio value in the presence of a 50 μM H_2_O_2_pulse and its corresponding basal ratio (Fig. 5B); and the recovery rate after the wash out stimulus of 500 μM H_2_O_2_, which reflects the intrinsic capacity of the intracellular environment to reduce disulfide bonds (Fig. 5C). As observed in Fig. 5A, analyzed cells display a wide range of basal values, from the keratinocytes cell line (CCD1102Kert) that shows a high baseline value reflecting a cytoplasmic environment adapted to a high oxidative tone, to the prostate carcinoma cell line (DU145), which seems to display a very low level of H_2_O_2_ at steady-state. This pattern is partially conserved in biosensor responses at 50 μM H_2_O_2_ (Fig. 5B), but is lost when cell types are compared in their recovery rates for the oxidized biosensor after removing the 500 μM H_2_O_2_pulse. In order to show that the recovery rate estimation can effectively discriminate between cell types, we used three cell lines: A549, which shows an intermediate basal signal but represents the highest recovery rate (1.35 ± 0.07 min^−1^); DU145, with the lowest basal signal and also a high recovery value (0.89 ± 0.09 min^−1^); and A704, which shows an intermediate basal signal and represents the lowest recovery value (0.28 ± 0.09 min^−1^) (Fig. 5D). These data allowed us to conclude that HyPer basal signal values are not associated to each other since basal peroxide levels are subject to diverse contributor processes balanced by the peroxide disposal system, while the HyPer signal recovery rate depends strictly on specific enzymatic activities involved in disulfide bond reduction.

### 3.6 Cell migratory capacity correlates with the recovery rate of the HyPer biosensor

**Fig. 6:**
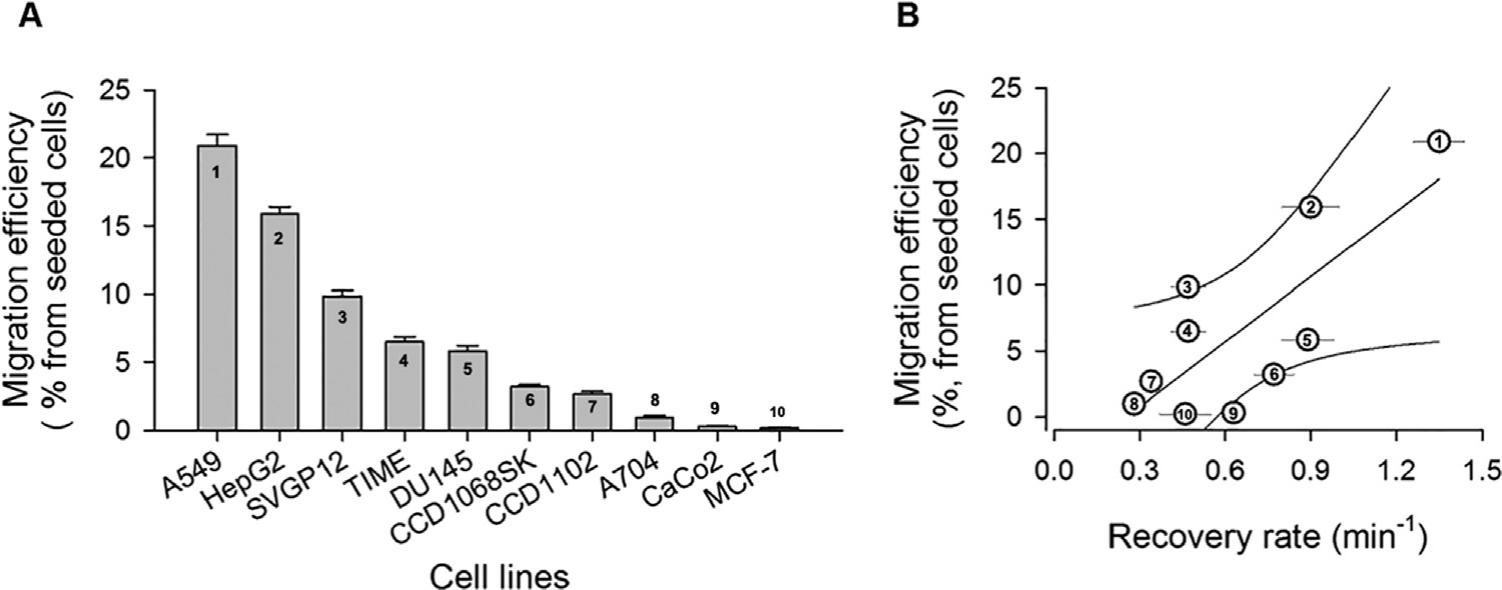
Migratory behavior of ten different cell lines correlates with HyPer signal recovery. A. Cells (6×10^4^) were plated in the upper compartment of a Transwell system and stimulated to migrate for 16 h under a 2% FBS stimulus. Migratory capacity was evaluated by counting the number of cells migrated per transwell and was expressed as a percentage of migration efficiency, relative to the initial number of seeded cells. Data are presented as averages±SE from at least three independent experiments. B) Correlation between migration efficiency and recovery rate of HyPer signal for each cell type (Pearson coefficient=0.77; p=.01).

Until now, our HyPer imaging data analysis determined the steady-state and kinetic values of HyPer, which are potentially useful to rank redox behaviors of cell types and the effectiveness of the treatments. However, whether this set of data is related to relevant cellular processes remains an open question. To answer this question, we explored whether HyPer recordings were able to indicate, via some of the measured parameters, a possible relationship with a cellular functional property associated with redox balance. To do that, we evaluated the migration capacity of ten cell lines in a double chamber system and ranked these cell lines according to their migration efficiency (Fig. 6A). The alignment of these data against HyPer parameters for each cell line produced statistically significant correlations with their recovery rates (Fig. 6B).

**Fig. 7:**
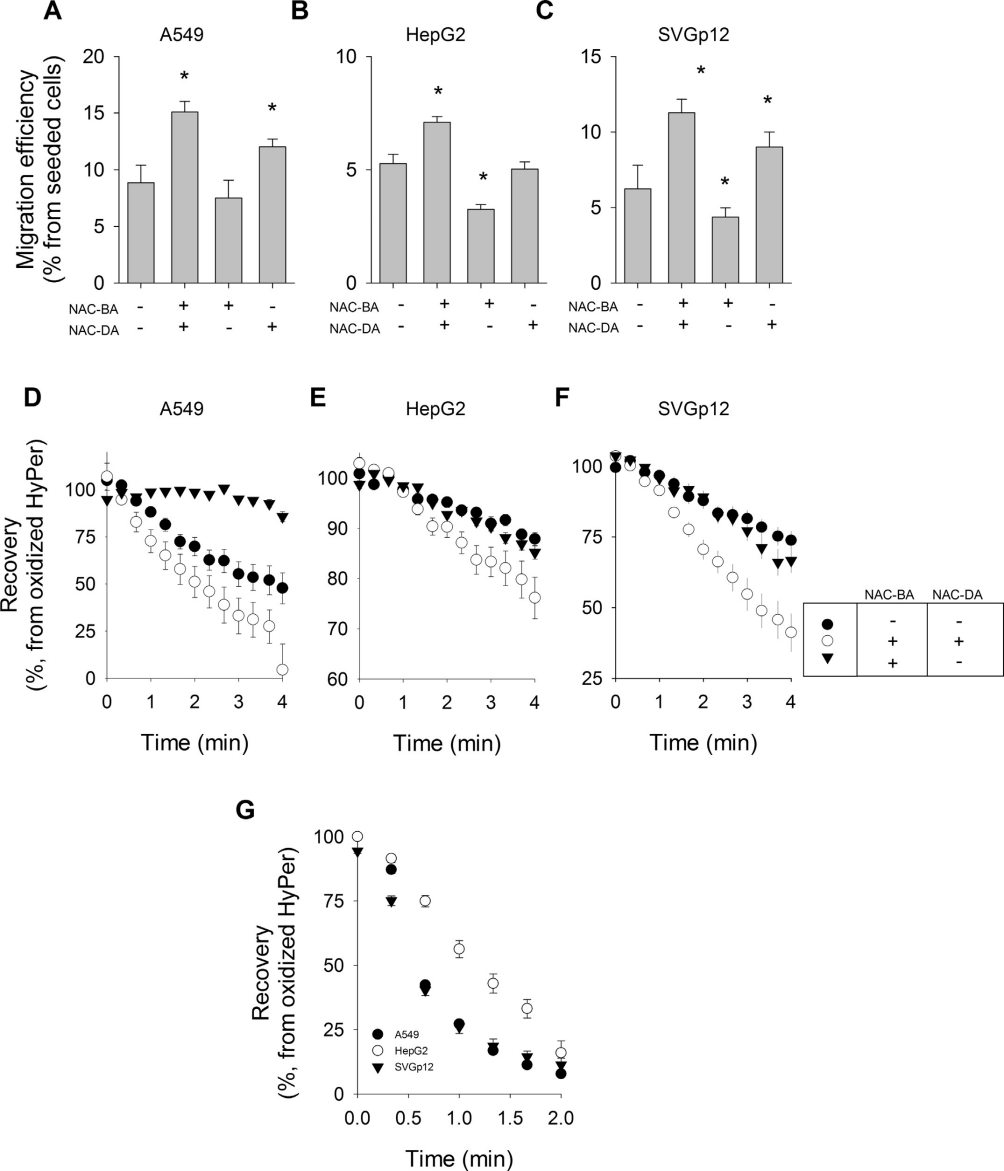
Treatment with N-Acetyl-Cysteine promotes migration capacity and accelerates the recovery of the HyPer signal biosensor in A549, HepG2 and SVGp12 cell lines. Migration assays were performed using (A) A549, (B) HepG2 and (C) SVGp12 cell lines. Treatment with 5 mM N-Acetyl-cysteine was administrated 12 h before the migration assay (NAC-BA) and during the migration assay (4 h) (NAC-DA). Data correspond to averages±SE of four independent assays and asterisks mean significant differences with respect to the control group (one-way ANOVA, Dunn's method for A and Bonferroni *t*-test for B and C). HyPer-expressing (D) A549, (E) HepG2 and (F) SVGp12 cells were treated with NAC as indicated by the box. Cells received a pulse of 500 μM H_2_O_2_ that completely oxidize the biosensor and increased the signal value to a maximum. After this, signal recovery was recorded, taken this value as the initial point. Experimental data from recovery curves for A549 cells were fitted to an exponential function: control cells (•), 66±13 s^−1^; NAC treated cells (○), 114±10 s^−1^; and wash out NAC (▼) 7±2 s^−1^. Likewise, estimated values for HepG2 were: control cells (•), 1.2 s^−1^, r=0.98; NAC treated (○) 1.9 s^−1^, r=0.99; wash out NAC (▼) 1.3 s^−1^, r=0.98). Recovery rates of SVGp12 cells were: control (•) 1.6 s^−1^, r=0.99; NAC treated (○) 3.7 s^−1^, r = 0.99; wash out NAC (▼) 1.1 s^−1^, r=0.91). All recovery curves were built from data from at least three independent experiments involving more than 10 recordings for each curve. G, HyPer signal recovery from A549 (•), HepG2 (○) and SVGp12 cells (▼) was obtained by removing the pulse of 500 μM H_2_O_2_ with a buffer KRH containing 5 mM NAC. Each recovery curve data correspond to averages±SE from at least 15 single cell recordings from a minimum of three independent experiments.

Additionally, three cells lines (A549, HepG2 and SVGp12) were chosen to evaluate the treatment effect of N-acetyl-cysteine (NAC, 5 mM) on their migration capacities. We considered a total period of 16 h that included 12 h of pre-incubation prior to the migration assay, which itself took 4 h. All three cell types responded increasing their migration efficiencies when exposed to NAC for the total period (16 h), compared with untreated cells (control) (Fig. 7A–C). This effect was shown to be dependent of the presence of NAC during the assay since despite treating cells for 12 h prior to conducting the migration assay in the absence of the cysteine donor, the levels of cellular migrations returned near to control conditions for A549 cells, and even lower for HepG2 and SVGp12 cells. Finally, we tested whether the presence of NAC during the migration assay was effective in promoting an increase in cellular motility. This was the case for A549 and SVGp12 cells, whose migration efficiencies were significantly higher than controls (Fig. 7A and C). In parallel, we evaluated if the treatments with NAC effectively accelerated the recovery rate of the biosensor in these three cell lines (Fig. 7D–F). For the three cell types treated with NAC, either over 16 continuous hours or only during H_2_O_2_ removal, we recorded accelerated recovery rates. In contrast, when NAC was removed for hours in order to simulate a migration assay without NAC, the recovery of the HyPer biosensor was similar to the control conditions.

## 4 Discussion

Our results demonstrate that the peroxide-sensitive biosensor HyPer constitutes a reliable tool for evaluating steady-state levels of protein oxidation by endogenous hydrogen peroxide and cellular reducing capacity in real-time through monitoring its recovery rates after oxidation. We targeted the HyPer biosensor to three different subcellular compartments that have very different redox conditions: the cytoplasm, the mitochondrion, and the endoplasmic reticulum. Our results indicate that recovery rates discriminate better the subcellular redox conditions than steady-state HyPer measurements (Fig. 2). Moreover, baseline values and H_2_O_2_ responses were largely affected by local pH. In contrast, the signal recovery of the biosensor assessed in the cytoplasm, which occurred spontaneously due to the activity of the cellular antioxidant machinery, was shown to be independent in experiments using pH clamping in living cells (Fig. 3).

We propose that the use of HyPer, a redox-responsible biosensor, overcomes the limitations of chemical-based techniques. The following attributes support the statement that HyPer is a reliable tool to evaluate antioxidant capacity in intact cells: (i) Fluctuations in the biosensor signal, either by the addition of exogenous peroxide or by spontaneous recovery, are obtained in a biologically relevant context. (ii) Ratiometric measurements reveal robust time- and dosage-dependent responses, avoiding the interference of fluorescence quenching and/or cellular volume changes, among other confounding factors. (iii) In addition to conventional steady-state measurements of HyPer, we consider that the kinetics of signal recovery offer a direct approach to determine the disulfide reduction activity in living cells, which comes to fulfill the need to test the biological impact of active biomolecules [20].

To gain insights into the nature of the biosensor behavior, we manipulated the activity of the Thioredoxin Reductase-Thioredoxin-1 axis by disrupting the assembly of seleno-proteins with auranofin, a selenium quelator [26] or by direct pharmacological inhibition of Thioredoxin-1 with PX-12. Both treatments hindered the cellular capacity to return the biosensor to a prior basal state (Fig. 4). In addition, incubating cells with EUK-134, a SOD-catalase mimetic, induced a decrease of hydrogen peroxide levels (Supplementary Fig. 4), as expected, but this treatment did not significantly affect the recovery rate of the biosensor (Fig. 4). These results reinforce the notion that the oxidation of cytosolic proteins and their respective disulfide reductions are a consequence of different activities of the redox machinery. Recently, Stöcker et al. reported a counterintuitive role for peroxiredoxins in transmitting the H_2_O_2_ equivalents to oxidation of thiol residues. By using mild and short hydrogen peroxide pulses, comparable to our experimental approach, this group demonstrated that protein oxidation induced by H_2_O_2_, and more specifically disulfide bond formation, depends mainly on the abundance of peroxiredoxins [27]. Here, we focused our investigation on the HyPer signal recovery as a reporter of reducing activity, which under our experimental settings was shown to depend at least partially on antioxidant seleno-enzymes and more directly on thioredoxin-1, but not by lowering endogenous H_2_O_2_ with EUK-134 (Fig. 4). These data support the idea that H_2_O_2_ diffusion and signaling rely on peroxiredoxins activity, while protein disulfide bond reduction is more a reflection of the activity of Thioredoxin Reductase-Thioredoxin-1 axis.

Cell migration is modulated by the availability of growth factors, chemokines, proteases and the extracellular matrix structure. In normal and tumoral cells, ROS production is also a relevant element of the intracellular machinery that allows cell migration [28]. Our results show that in HyPer-expressing cells, migration efficiency partially correlates with the recovery rate of the HyPer signal, a functional expression of the intracellular reduction capacity (Fig. 6). Cell migration is essentially an asymmetric phenomenon in which the migratory front and rear respond in a different manner to migratory stimulus. Migration front, characterized by cellular protrusions, presents higher levels of hydrogen peroxide than cell body. In this specialized structure, H_2_O_2_ oxidizes cysteine residues of cofilin, which in this state, binds G-actin and modulates actin dynamics [29]. Plasma membrane located at the migration front in HeLa and PC3 cells, both cancer cell lines, are provided with TRPM2 channels that sense local H_2_O_2_ and allow Ca^2+^ influx and accumulation of Zn^2+^ at this region, which according to the authors represents a novel role for Zn^2+^ in cellular migration that antagonizes the well-known role of Ca^2+^ [30]. The role of redox potential in the rear zone has been less characterized. However, it has been demonstrated that the activity of calpain, protease that plays an important role in the migratory machinery at the rear zone [31], is inhibited in a reversible manner by H_2_O_2_-induced disulfide bond formation. All these pieces of evidence depict a functional uneven distribution of redox agents in moving cells. HyPer measurements presented in this work were taken from the bulk of standing cells and therefore, we cannot be able to assign a specific role to redox zoning. This experimental limitation, joined to the complexity of the migratory processes, might the reflection of the weak correlation between recovery rates and migration efficiency that we found. Nevertheless, improving the targeting of the biosensor to the plasma membrane and measuring recovery rates at the rear zone could unveil a notorious redox front-to-rear gradient in living cells subjected to a migratory stimulus.

We also established that living cells presented high variability in redox properties, even though most of human cell lines we used had a carcinogenic origin. In addition to this complexity, we observed that NAC treatment had effect on migratory efficiency only when it was present during the migratory assay (Fig. 7). Removal of NAC from migration assay and biosensor recovery experiments totally reversed the original effects, suggesting that redox organization inside the cells is a time-dependent property, which is not only defined by local H_2_O_2_ production, but also by corresponding enzymatic disulfide reduction, which are necessary for modulating H_2_O_2_-triggered oxidation processes at these microdomains. Therefore, expected effects for a given antioxidant input must be taken cautiously, considering not only the temporal application mode but also the phenotype of the cell/tissue target.

## 5 Conclusions

• HyPer biosensor kinetic represent a reliable tool to measure intracellular redox activity and to evaluate the impact of exogenous molecules with potential antioxidant properties.
• Recovery rates of HyPer signal after an H_2_O_2_ challenge report the reductive tone of the environment, where the biosensor is located in a pH-independent manner.
• Cellular migration efficiency responds to the reductive machinery at the cytoplasm, which in turn can be modified by exogenous compounds.

## Funding

This work was supported by the Chile's National Commission for Scientific and Technological Research, CONICYT [FONDECYT 1120201, OP] [FONDECYT 1160900, DV] [FONDEF CA13I10013/IT15I10048, OP]. The Millennium Nucleus of Ion Channels-Associated Diseases (MiNICAD) is a Millennium Nucleus supported by the Iniciativa Científica Milenio of the Ministry of Economy, Development and Tourism (Chile).

## Declaration of interest

None.

## Appendix A Supplementary material

Supplementary Fig. 1. HyPer signal response in EOMA cells upon exogenous H_2_O_2_. A. Time-course of HyPer fluorescence obtained from a single TIME cell excited at 420 nm (purple trace) and at 490 nm (green trace). Addition of exogenous hydrogen peroxide (50 and 500 μM) is marked by dotted lines within the plots. B. An example of HyPer recording; data is expressed as the ratio between the 420 and 490 nm signals. To facilitate data visualization, experimental data of the bin used to determinate basal values (basal, green) were used; points assigned to signal increase at 50 μM H_2_O_2_ pulse are in yellow; points belonging to the plateau are submaximal (orange); maximal value (Max) is obtained by applying 500 μM H_2_O_2_ pulse; averaging experimental data colored in red. Finally, spontaneous recovery of biosensor signal was quantified from points obtained after peroxide pulse removal, in green. C. Dose-response curve built from TIME cells exposed to the H_2_O_2_ concentration indicated in the plot. Each experimental point corresponds to averages±SE of several ratio values obtained from 9 to 28 cells from at least three independent experiments for each concentration evaluated. Data were fitted to a four-parameter logistic function (r=0.987, dotted line), which served to estimate an EC_50_=42±5 μM.

Supplementary Fig. 2. Subcellular expression of HyPer biosensor in AdHek cells. In photos **A**, **B** and **C**, Ad-293 cells were transfected with a plasmid carrying the HyPer biosensor targeting the endoplasmic reticulum. Cells are presented at (A) transmitted light, (B) emitted light at 520 nm, corresponding to HyPer and (C) a merged photo with DAPI staining to visualize nuclei. In the middle, cytoplasmic expression of HyPer is depicted under (D) a bright field, (E) HyPer fluorescence and (F) DAPI staining merged with biosensor fluorescence. White bar on B image represents ten micrometers and it is valid for A-F images. At the bottom, mitochondrial expression of HyPer is shown, with cells visualized under (G) a bright field, (H) biosensor fluorescence and (I) DAPI staining merged with biosensor fluorescence. In A and G, cellular contours were drawn with a dotted red line to facilitate visual localization of the biosensor. White bar on G image represents five micrometers, which is valid for G-I images.

Supplementary Fig. 3. pH clamp in living cells. A. TIME cells loaded with BCECF were exposed to a mixture of nigericin/valinomycin ionophores (100 μM/25 μM), as indicated by the white bar and dotted line. Extracellular media was then replaced by a high K^+^-KRH, as indicated by the black bar at several adjusted pH values; numbers on the trace indicate the pH of the buffer. B. A calibration curve was built with BCECF ratios obtained at the defined pH values. Data were fitted to a linear regression (dotted line). Data correspond to averages ± SE of 22 cells from three independent experiments. C. Ad-293 cells expressing cytosolic SyPher biosensor were exposed to the same mixture of ionophores as in A and subjected to a high K^+^ buffer, adjusted to the indicated pH values. The trace showed in the graph corresponds to the average±SE of SyPher ratio from 13 cells from one representative experiment. D**.** A calibration curve built with SyPher ratios plotted as a function of imposed pH values. Data obtained from three independent experiments correspond to averages±SE of 38 cells from three independent experiments. Data were fitted to a linear regression (dotted line).

Supplementary Fig. 4. Effect of Auranofin and EUK-134 on HyPer baseline values in TIME cells. A. HyPer-expressing TIME cells were pre-incubated with auranofin for 24 h at the concentrations indicated. Data show averages±SE from the control group of 74 cells from six independent experiments; the 10 nM and 100 nM auranofin groups were 29 cells and 26 cells, respectively, both collected from three independent experiments. **B**. HyPer basal values of groups of TIME cells exposed to EUK-134 for 24 h or untreated (control), presented as averages ± SE. The control group includes 30 cells from four independent experiments; the 100 nM EUK-134 group consisted of 27 cells from three independent experiments; the 1 μM EUK-134 group consisted of 37 cells from four independent experiments and the 10 μM EUK-134 group consisted of 32 cells from three independent experiments.
