# Supplementary figures and images for: Insights into the HyPer biosensor as molecular tool for monitoring cellular antioxidant capacity
Source: Redox Biol. 2018 Mar 2;16:199–208. doi: 10.1016/j.redox.2018.02.023 (PMC5952670; doi:10.1016/j.redox.2018.02.023)

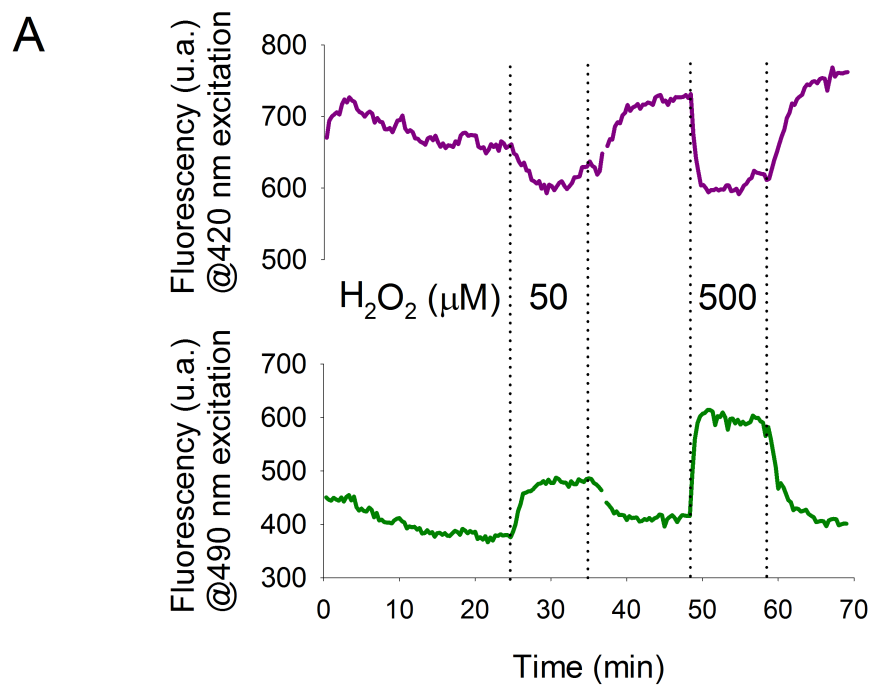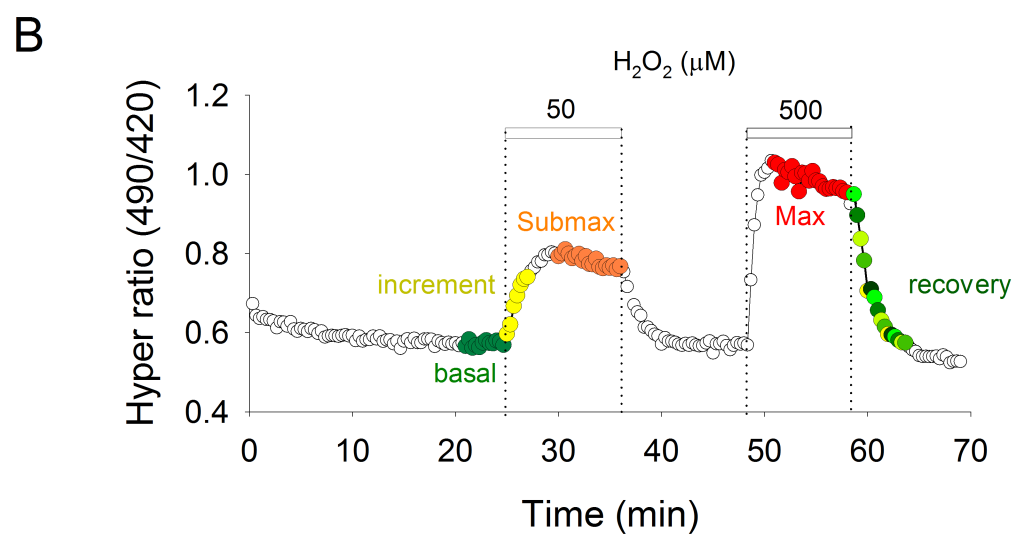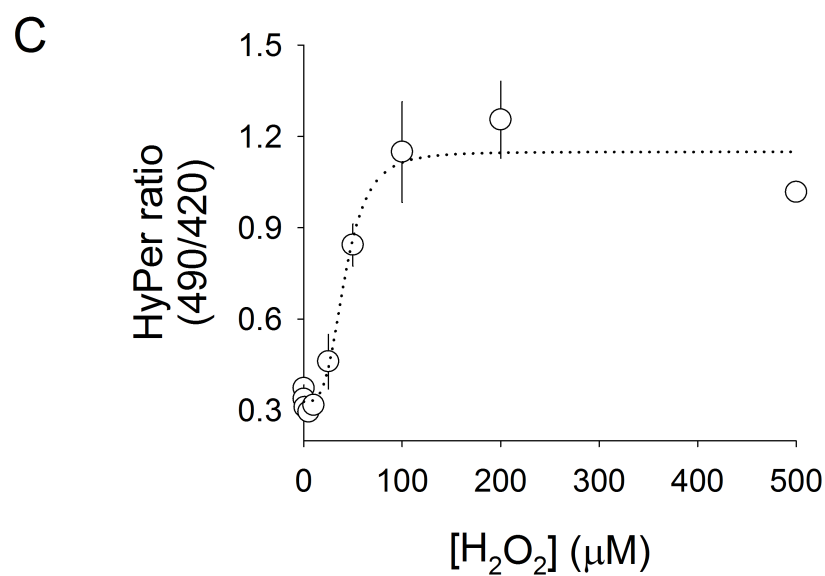

Supplement: Supplementary file 1 — Supplementary Fig. 1. HyPer signal response in EOMA cells upon exogenous H2O2. A. Time-course of HyPer fluorescence obtained from a single TIME cell excited at 420 nm (purple trace) and at 490 nm (green trace). Addition of exogenous hydrogen peroxide (50 and 500 μM) is marked by dotted lines within the plots. B. An example of HyPer recording; data is expressed as the ratio between the 420 and 490 nm signals. To facilitate data visualization, experimental data of the bin used to determinate basal values (basal, green) were used; points assigned to signal increase at 50 μM H2O2 pulse are in yellow; points belonging to the plateau are submaximal (orange); maximal value (Max) is obtained by applying 500 μM H2O2 pulse; averaging experimental data colored in red. Finally, spontaneous recovery of biosensor signal was quantified from points obtained after peroxide pulse removal, in green. C. Dose-response curve built from TIME cells exposed to the H2O2 concentration indicated in the plot. Each experimental point corresponds to averages±SE of several ratio values obtained from 9 to 28 cells from at least three independent experiments for each concentration evaluated. Data were fitted to a four-parameter logistic function (r=0.987, dotted line), which served to estimate an EC50=42±5 μM. [file mmc1.pdf]

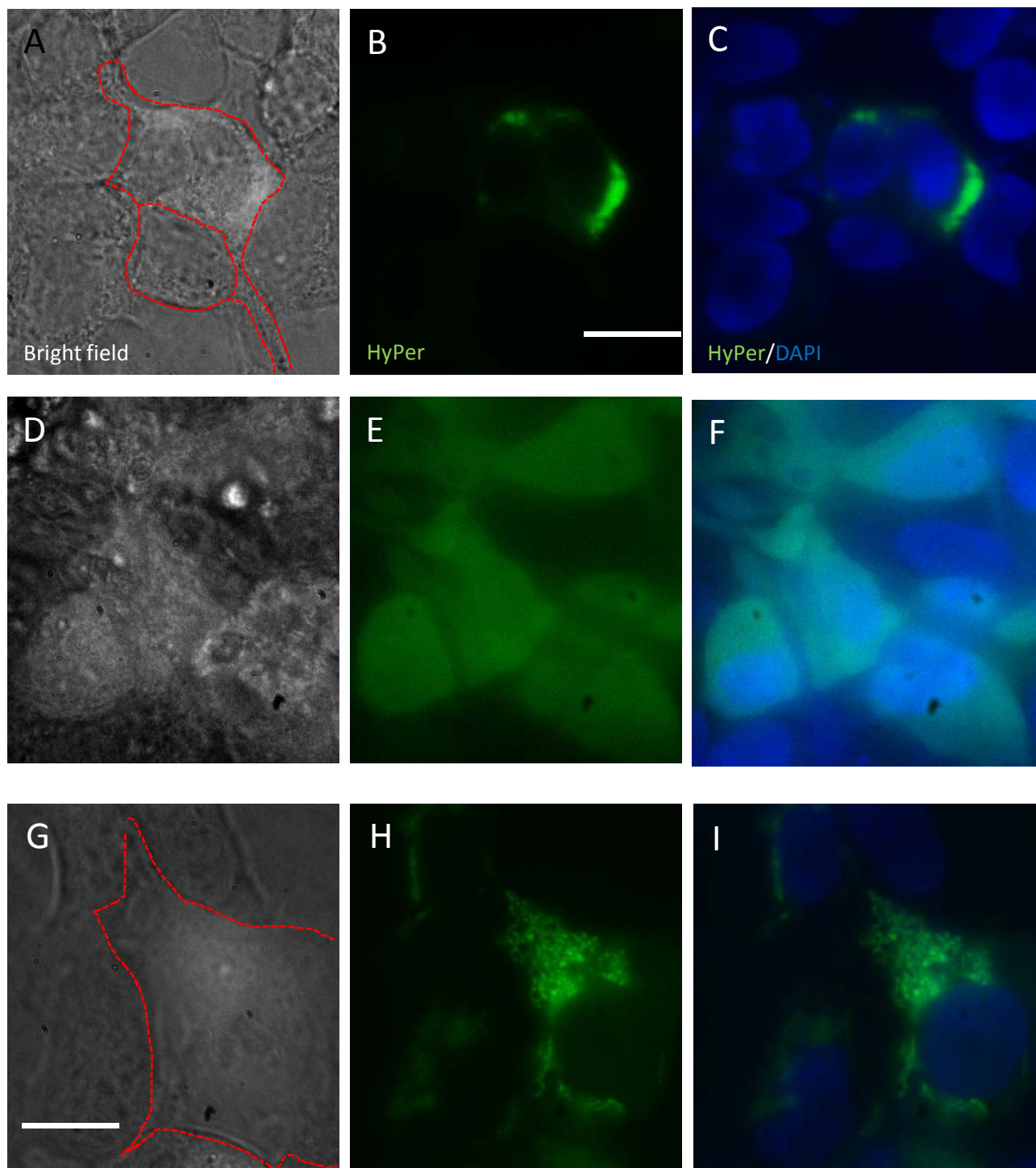

Supplementary figure 2

Supplement: Supplementary file 2 — Supplementary Fig. 2. Subcellular expression of HyPer biosensor in AdHek cells. In photos A, B and C, Ad-293 cells were transfected with a plasmid carrying the HyPer biosensor targeting the endoplasmic reticulum. Cells are presented at (A) transmitted light, (B) emitted light at 520 nm, corresponding to HyPer and (C) a merged photo with DAPI staining to visualize nuclei. In the middle, cytoplasmic expression of HyPer is depicted under (D) a bright field, (E) HyPer fluorescence and (F) DAPI staining merged with biosensor fluorescence. White bar on B image represents ten micrometers and it is valid for A-F images. At the bottom, mitochondrial expression of HyPer is shown, with cells visualized under (G) a bright field, (H) biosensor fluorescence and (I) DAPI staining merged with biosensor fluorescence. In A and G, cellular contours were drawn with a dotted red line to facilitate visual localization of the biosensor. White bar on G image represents five micrometers, which is valid for G-I images. [file mmc2.pdf]

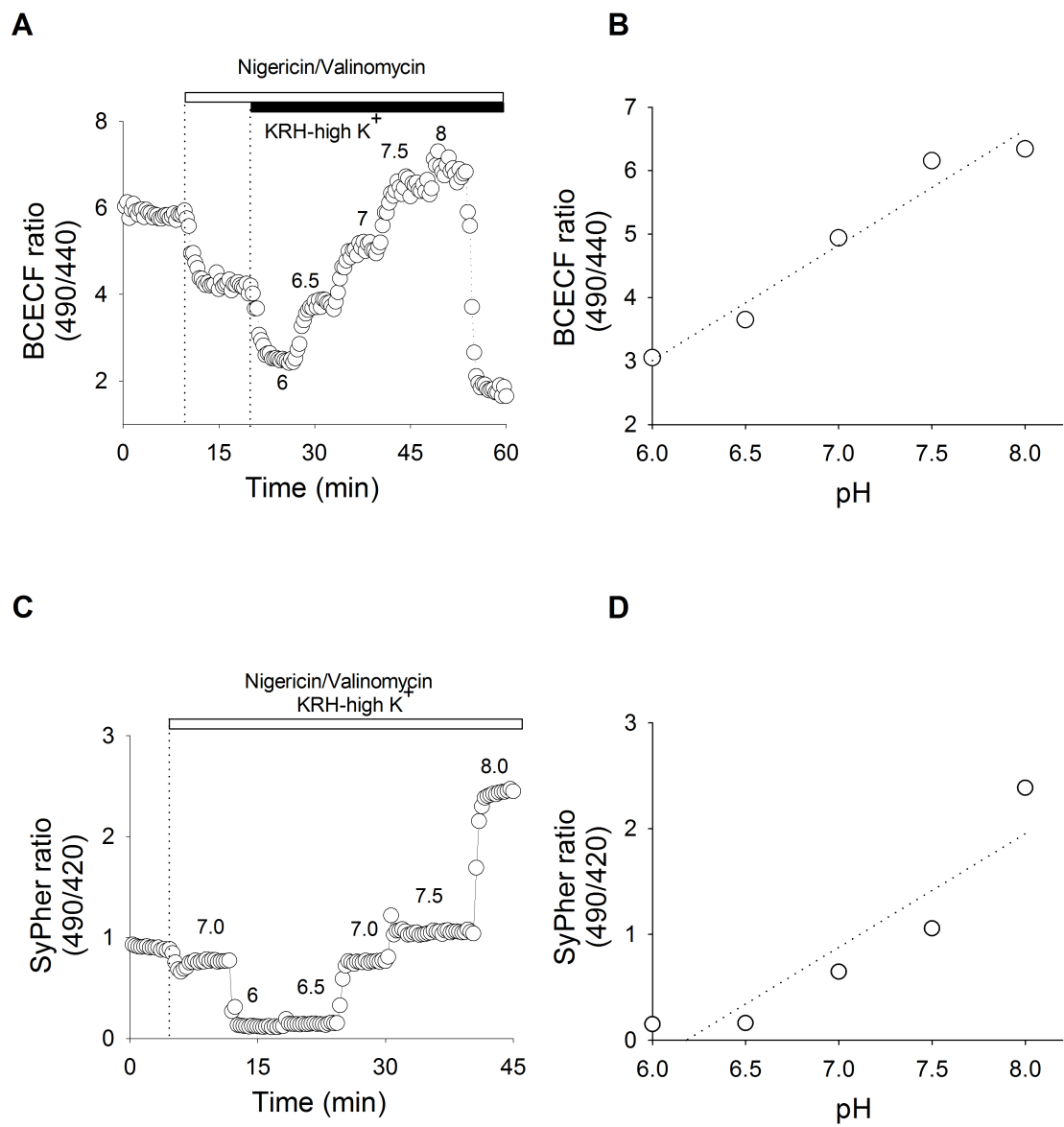

Supplementary figure 3

Supplement: Supplementary file 3 — Supplementary Fig. 3. pH clamp in living cells. A. TIME cells loaded with BCECF were exposed to a mixture of nigericin/valinomycin ionophores (100 μM/25 μM), as indicated by the white bar and dotted line. Extracellular media was then replaced by a high K+-KRH, as indicated by the black bar at several adjusted pH values; numbers on the trace indicate the pH of the buffer. B. A calibration curve was built with BCECF ratios obtained at the defined pH values. Data were fitted to a linear regression (dotted line). Data correspond to averages ± SE of 22 cells from three independent experiments. C. Ad-293 cells expressing cytosolic SyPher biosensor were exposed to the same mixture of ionophores as in A and subjected to a high K+ buffer, adjusted to the indicated pH values. The trace showed in the graph corresponds to the average±SE of SyPher ratio from 13 cells from one representative experiment. D. A calibration curve built with SyPher ratios plotted as a function of imposed pH values. Data obtained from three independent experiments correspond to averages±SE of 38 cells from three independent experiments. Data were fitted to a linear regression (dotted line). [file mmc3.pdf]

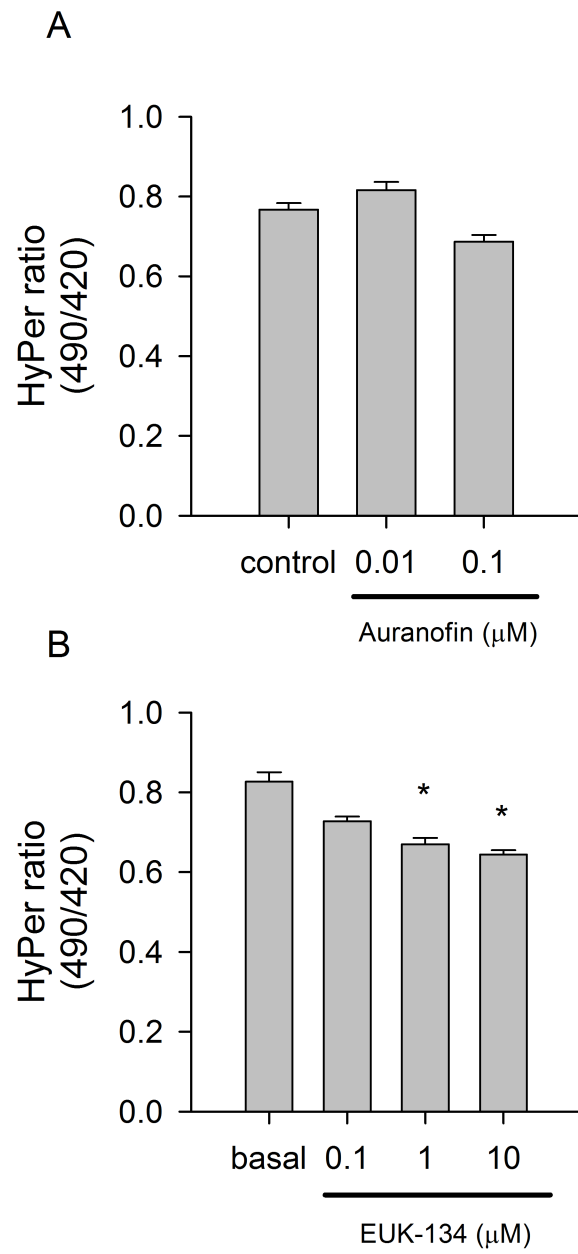

Supplementary figure 4

Supplement: Supplementary file 4 — Supplementary Fig. 4. Effect of Auranofin and EUK-134 on HyPer baseline values in TIME cells. A. HyPer-expressing TIME cells were pre-incubated with auranofin for 24 h at the concentrations indicated. Data show averages±SE from the control group of 74 cells from six independent experiments; the 10 nM and 100 nM auranofin groups were 29 cells and 26 cells, respectively, both collected from three independent experiments. B. HyPer basal values of groups of TIME cells exposed to EUK-134 for 24 h or untreated (control), presented as averages ± SE. The control group includes 30 cells from four independent experiments; the 100 nM EUK-134 group consisted of 27 cells from three independent experiments; the 1 μM EUK-134 group consisted of 37 cells from four independent experiments and the 10 μM EUK-134 group consisted of 32 cells from three independent experiments. [file mmc4.pdf]
